# Drug Injection-Related and Sexual Behavior Changes in Drug Injecting Networks after the Transmission Reduction Intervention Project (TRIP): A Social Network-Based Study in Athens, Greece

**DOI:** 10.3390/ijerph18052388

**Published:** 2021-03-01

**Authors:** George Giallouros, Katerina Pantavou, Despina Pampaka, Eirini Pavlitina, Daniele Piovani, Stefanos Bonovas, Georgios K. Nikolopoulos

**Affiliations:** 1 Department of Business and Public Administration, University of Cyprus, Nicosia 1678, Cyprus; giallouros.giorgos@ucy.ac.cy; 2 Medical School, University of Cyprus, Nicosia 2029, Cyprus; pantavou.katerina@ucy.ac.cy; 3 Cyprus International Institute for Environmental and Public Health, Cyprus University of Technology, Limassol 3041, Cyprus; despina.pampaka@cut.ac.cy; 4 Transmission Reduction Intervention Project, Athens Site, 11527 Athens, Greece; pavlitina.eirini@gmail.com; 5 Department of Biomedical Sciences, Humanitas University, 20090 Milan, Italy; dpiovani@hotmail.com; 6 IRCCS Humanitas Research Hospital, 20089 Milan, Italy

**Keywords:** PWID, recent infection, HIV, networks, injecting-related behaviors, sexual behaviors

## Abstract

The Transmission Reduction Intervention Project (TRIP) was a network-based, enhanced contact tracing approach, targeting recently HIV-infected people who inject drugs (PWID) in Athens, Greece (2013–2015). This analysis examines behavioral changes of participants in TRIP and their determinants between baseline and follow-up visits to the program. All participants of TRIP were tested for HIV and interviewed using a questionnaire with items on drug injection-related and sexual behaviors. Multivariable logistic regression models were used to examine potential relationships between participants’ behaviors and sociodemographic or other characteristics. The analysis included 292 participants. At follow-up, the percentage of participants who injected drugs decreased [92.5%, *n* = 270 versus 72.3%, *n* = 211 (*p* < 0.001)], and more participants adopted safer behaviors. Employment, age, and gender were significantly associated with some behavioral changes. For instance, unemployed participants were half as likely as the employed to stop drug injection [adjusted odds ratio (aOR): 0.475, 95% confidence interval (CI): 0.228, 0.988]. Increasing age was associated with lower probability of sharing syringes at follow-up (aOR: 0.936, 95%CI: 0.887, 0.988). Finally, females were less likely than males to improve their behavior related to sharing cookers, filters, or rinse water (aOR: 0.273, 95% CI: 0.100, 0.745). In conclusion, adoption of safer behaviors was observed following TRIP implementation. Future prevention programs should focus on younger PWID and especially females. Social efforts to support employment of PWID are also important.

## 1. Introduction

The global health burden from human immunodeficiency virus (HIV) remains high: at the end of 2019, 38 million [31.6–44.5 million] people were living with HIV (PLHIV), and 1.7 million individuals became infected [1]. Without treatment, PLHIV may develop AIDS and experience opportunistic infections and cancers. Nowadays, however, PLHIV, who are on combination antiretroviral treatment (ART), have a similar life expectancy to individuals without HIV and they are consequently at increased risk of developing non-AIDS comorbidities such as cardiovascular disease [2]. As an effective vaccine for eradication of HIV has not yet been developed [3], preventive measures for the containment of the virus remain to date the first public health priority.

Use of condoms, when practiced consistently and appropriately, reduce HIV incidence [4]. Effective prevention tools also include male circumcision [5], and ART for prevention or as a pre-exposure prophylaxis (PREP) in HIV-negative individuals [6,7,8,9]. Among people who inject drugs (PWID), harm reduction including needle and syringe programs (NSPs) and opioid substitution therapy (OST) is very important. NSPs supply PWID with sterile needles and syringes and are considered an essential component of HIV prevention amongst PWID [10]. OST refers to administering PWID a replacement, i.e., a prescribed medicine, such as methadone or buprenorphine, which is usually taken orally in a supervised clinical setting. OST, especially when adherence is high, has been proven to substantially reduce injection drug use and HIV transmission [11]. There is no single prevention tool for optimal suppression and containment of HIV. Instead, implementation of multidisciplinary programs is considered more effective in tackling holistically the spread of HIV [12,13]. 

In Greece, the majority of HIV infections were primarily among men who have sex with men (MSM). However, an outbreak of HIV among PWID was observed in 2011–2013, in the context of the Greek financial crisis of 2010, which probably influenced the size of drug injecting networks and normative behaviors in them [14,15,16,17]. The ground for HIV transmission was fertile, even before the financial crisis, given the suboptimal development and implementation of harm reduction programs [18]. While the outbreak had been leveling off, a network-based program, the Transmission Reduction Intervention Project (TRIP), was conducted targeting individuals recently infected with HIV and their networks’ associates. 

The aim of the present study was to identify factors associated with changes in drug injection-related and sexual behaviors amongst PWID and their networks in Athens, Greece, following the implementation of TRIP, a social network-based project, in the context of a large HIV outbreak following a big economic crisis. 

## 2. Materials and Methods

### 2.1. Study Population

TRIP was conducted in Athens, Greece, between June 2013 and July 2015 [19]. The intervention was based on tracing the networks of HIV-infected people, especially those who were recently infected (in the past 6 months). The concept of TRIP was that if someone has recently acquired HIV, it is likely that the person who infected him/her and those whom the infected person has infected are in the same network. Recruiting network members of recently HIV-infected people is thus likely to identify more people who also got HIV recently and, therefore, could be highly infectious [19].

Those eligible to participate were individuals older than 18 years old, who lived in Athens (capital city of Greece) in the past 12 months, spoke Greek or English, and consented to the laboratory tests and to all the procedures of the study. Some HIV seropositive individuals were referred to TRIP by collaborating testing sites. All TRIP participants were interviewed face-to-face by experienced personnel using a structured questionnaire and tested for HIV and recent infection (if they were HIV-positive) at baseline. Recent infection was detected using limiting antigen avidity (LAg) assay (Sedia^TM^ Biosciences Corporation). One follow-up (interview and sample collection) was scheduled at 6 months after enrolment [19].

Based on HIV status, testing history, and the results of LAg, TRIP participants were classified into five groups. The first group consisted of recent seeds (RS), who were newly HIV-diagnosed PWID referred from collaborating testing facilities and were probably recently infected. The second (comparison) group of seeds consisted of participants who were newly HIV-diagnosed but probably not with a recent infection, namely control seeds with long-term HIV infection (LCS). Seed is a term used to refer to a primary participant whose network was traced. Thus, RS and LCS were asked to help recruit and test members of their social networks. HIV-negative individuals at baseline (negative controls without tracing in their networks) were also enrolled. Overall, the participants were classified into the following five groups: (a) RS, (b) LCS, (c) network members of RS, (d) network members of LCS, and (e) HIV-negative controls.

TRIP had many elements of partner services as suggested by the United States (US) Centers for Disease Control and Prevention (CDC) [20]. The recently HIV-infected recruits were supported by project staff to get in contact with care or stay in contact with care. Additionally, recently HIV-infected were given regularly counseling. Apart from the recently-HIV infected people who were prioritized, health education and other support were provided to all participants. Strategies to facilitate linkage and retention to care were adopted: (a) information and support were distributed on where the facilities/services are and how to contact them; (b) support was provided by project staff in arranging appointments and at some instances participants were accompanied to the clinics or other facilities; (c) signaling participants by telephoning or sending them text messages to remind them of appointments or medical exams; and (d) facilitating administrative procedures for the improvement of the general wellbeing of participants by assisting them in obtaining legal documents, getting health insurance and benefits, finding employment, and receiving legal advice [21].

### 2.2. Behavioral Data

The focus of this work was to assess drug injection-related and sexual behavior trends of TRIP participants between baseline and follow-up (6 months apart). In the analysis, we included only data from respondents that participated in both rounds (baseline and follow-up) of TRIP.

The TRIP questionnaire items (Q) and sample responses (R) are presented in a Supplementary Materials (Table S1). At first, the participants were asked to report their drug injection status, i.e., whether they had injected any drugs or had someone other than an accredited professional inject them with any drugs (Q1) during the past six months. The items followed were related to participants’ drug injection-related (Q2–Q4) and sexual behaviors (Q5-Q8) considering also the past six months of the interview. In particular, the participants were asked to report:

- the number of different people they injected drugs with (Q2),
- how often they injected drugs (Q3),
- the proportion of the time that they had shared or given to someone injection equipment (syringe, a cooker, filter or rinse water) or they had backloaded (piggy-back) to share injection drugs (Q4a–Q4e),
- the number of people they had sex with (Q5 and Q7) and
- the number of people they had sex with and always used a condom (Q6 and Q8).

The responses were dichotomous (yes; no) (R1), open-ended to define the number of drug injecting or sexual partners (R2, R5-R8), and nine or ten-point rating scales (R3, R4) lingually describing the number of occasions (never; only a few times; 1–3 times/month; about once/week; 2–5 times/week; about once/day; 2 to 3 times/day, almost every day; 4 to 9 times/day, almost every day; 10+ times/day, almost every day) or the proportion of the time (none of the time; very little; less than half; about half; more than half; almost all; all; not applicable; not asked; does not know) that participants had been involved in a behavior.

### 2.3. Statistical Analysis

Continuous variables were described using median and mean values with interquartile ranges and standard deviations, while categorical variables using frequencies and percentages. The chi-squared test was used to examine potential differences in behavior across sociodemographic characteristics. The comparison between responses to questionnaire items at baseline and follow-up was performed using paired-data statistical methods. For cases where either a baseline or follow-up response was missing, the participant was excluded from the paired analysis. Exact McNemar’s test was used for dichotomous categorical variables and Bowker’s test for table symmetry [22] in the case of categorical variables with more than two response categories. The matched sign-rank test was used for the discrete variables.

The responses to questionnaire item Q2 were analyzed as a discrete variable. In order to gain statistical power, responses to Q3 were merged into three categories encompassed by rare (never; only a few times; 1–3 times/month), weekly (about once/week; 2–5 times/week), and daily (about once/day; 2 to 3 times/day, almost every day; 4 to 9 times/day, almost every day; 10+ times/day, almost every day) injection of drugs. The responses to Q4 were collapsed into two categories, safe (none of the time) versus risky (very little; less than half; about half; more than half; almost all; all) behavior.

The responses in questionnaire items regarding sexual behavior, Q5–Q8, were combined to illustrate the proportion of sex partners with whom a condom was always used, separately for men and women. Therefore, for men, the number of female sex partners whom they always used a condom with, in Q6, was divided by the total number of female sex partners of Q5 (Figure S1). Similarly, for women, the number of male sex partners whom they always used a condom with, in Q8, was divided by the total number of male sex partners of Q7. The proportions calculated illustrate the sex partners’ proportion each participant had safe sex with and was analyzed using sign rank test to examine the differences at baseline versus follow-up. All baseline versus follow-up comparisons were repeated independently for the different participant groups (RS, LCS, network of RS, network of LCS and negative controls), and also for HIV status (negative and positive).

Regression analysis involved the application of various logistic models to identify predictors of risky behaviors at the follow-up visit as they were exemplified in the questionnaire responses. Univariable logistic regression models were first considered, with significant independent predictors (*p* < 0.05) consequently included in the multivariable logistic regression models. The participant group was considered in multivariable models independently of univariable analysis results due to the physical significance of the variable. Responses at the follow-up were regressed against different variables after controlling for the response scoring at baseline. Binary responses, R1 and R4 were regressed using binary logistic regression, while the R3 multi-categorical response variable was regressed using ordered categorical logistic regression. The sexual behavior proportions were regressed using fractional outcome regression models, suitable for a dependent variable that is greater than or equal to 0 and less than or equal to 1. Similarly, we carried an analysis to identify predictors for a behavioral improvement at follow-up versus baseline. This was performed by modeling a binary coded variable for improvement or not, against the specified predictors. A *p* value less than 0.05 signified statistical significance. All statistical analyses were conducted in Stata v. 14 (Stata Corp., Spring Valley, MN, USA).

### 2.4. Ethical Statement

The intervention (DP1DA034989—ClinicalTrials.gov identifier: NCT01827228) was approved by the Institutional Review Boards of the National Development and Research Institutes (NDRI) in New York City (IRB00000634—April 2013) and of the Hellenic Scientific Society for the study of AIDS and Sexually Transmitted Diseases in Athens, Greece (IRB00002095—May 2013). All experiments were performed in accordance with relevant guidelines and regulations. All participants provided written informed consent.

## 3. Results

### 3.1. Socio-Demographic Characteristics of Participants

The 81.8% (*n* = 292 out of 357) of the participants who were recruited at baseline were followed-up after a period of six months (Table S2). The sociodemographic characteristics of the participants who were followed-up were significantly different compared to those who were lost to follow-up with regards to homelessness status (homeless 19.2% among those followed up vs. 38.5% among those lost to follow-up, *p* < 0.001), nationality (non-Greeks 7.9% among those followed up vs. 16.9% among those lost to follow-up, *p* = 0.025) and participant group (network of RS, 46.6% among those followed up vs. 53.9% among those lost to follow-up, *p* = 0.030).

The sociodemographic characteristics of the 292 participants who underwent both the baseline and follow-up assessments of TRIP are presented in Table 1. The participants were mainly males (*n* = 231, 79.1%), with an overall median age of 35 years (IQR 31–41 years). Most of the participants were of Greek nationality (*n* = 269, 92.1%), while those of non-Greek nationality (*n* = 23, 7.9%) were mainly from countries in the Middle East or Africa. Only 14.4% (*n* = 42) had received post-high school education, 20.5% (*n* = 60) were employed, whereas 13.4% (*n* = 39) were homeless at follow-up. The distribution of participants amongst the different groups varied with 22 (7.5%) of them classified as RS, 17 (5.8%) LCS, 136 (46.6%) network members of RS, 47 (16.1%) network members of LCS, and 70 (24%) HIV-negative controls. As expected, the prevalence of HIV among participants was high with 122 (41.8%) being HIV-positive and 170 (58.2%) negative (*p* = 0.031).

### 3.2. Drug Injection Status

**Figure 1:**
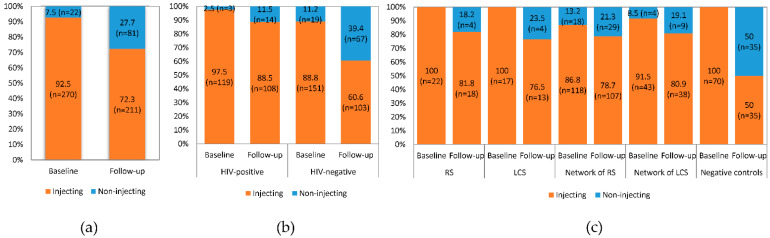
Frequencies of the responses to questionnaire item Q1 (Have you injected drugs, or had someone other than an accredited professional inject you with any drugs?) (*n* = 292), at baseline and follow-up of Transmission Reduction Intervention Project (TRIP) (**a**) all participants, (**b**) by human immunodeficiency virus (HIV) status, and (**c**) by participant group. Abbreviations: RS, recent seeds; LCS, control seeds with long-term HIV infection.

Τhe percentage of participants who injected drugs or had someone other than an accredited professional inject them with drugs at baseline decreased significantly at follow-up (92.5%, *n* = 270 versus 72.3%, *n* = 211; *p* < 0.001) (Figure 1a). This was evident regardless of participants’ HIV status (Figure 1b) or participant group (Figure 1c). Statistical significance was reached in both HIV-positive (*p* = 0.002) and HIV-negative (*p* < 0.001) participants while in the case of participant groups, statistical significance was reached in the network of RS (*p* = 0.027) and the negative controls (*p* < 0.001) (Table S3).

Sixty-five out of the 270 (22.3%) PWID stopped and six out of 22 participants started to inject drugs at follow-up (Table S3). The percentage of employed PWID who stopped injecting drugs at follow-up (40.4%, *n* = 19) was about twice that of the unemployed (20.6%, *n* = 46; *p* = 0.004) (Supplementary Materials, Table S4). Moreover, HIV-negative PWID stopped injecting drugs at follow-up at a higher rate than HIV-positive PWID. This was evident both in the analysis by HIV status (stopped 35.1%, *n* = 53 HIV-negative versus 10.1%, 12 HIV-positive) and by participant group (stopped 50.0%, 35 network controls).

Multivariable binary logistic regression showed that injecting drugs at follow-up was significantly related to whether or not a participant injected drugs at baseline (adjusted Odds Ratio—aOR: 10.576, 95% CI: 3.346–33.433), as expected (Supplementary Materials, Table S5). Males and unemployed participants were about two times more likely to have injected drugs during the past six months in follow-up (aOR: 0.399, 95% CI: 0.195, 0.819 for females versus males and aOR: 2.706, 95% CI: 1.332, 5.495 for unemployed versus employed) than females and employed participants. Recent seeds, network of RS and of LCS were also more likely to inject drugs at follow-up compared to negative controls (aOR: 4.420, 95% CI: 1.298, 15.052; aOR: 4.903, 95% CI: 2.348, 10.239; aOR: 5.369, 95% CI: 1.965, 14.671).

Although in univariable analysis age and HIV status were statistically significantly related to stopping drug injection at follow-up, multivariable logistic regression analysis produced significant results only for employment status and participant group (Table S5). Employed participants were twice as likely as the unemployed to stop drug injection at follow-up (aOR: 0.475, 95% CI: 0.228, 0.988 for unemployed versus employed). Moreover, RS (aOR: 0.233, 95% CI: 0.070, 0.778), network members of RS (aOR: 0.186, 95% CI: 0.090, 0.382) and network members of LCS (aOR: 0.185, 95% CI: 0.068, 0.502) were four times less likely to stop drug injection at follow-up compared to negative controls.

### 3.3. Drug Injection-Related Behaviors

**Figure 2:**
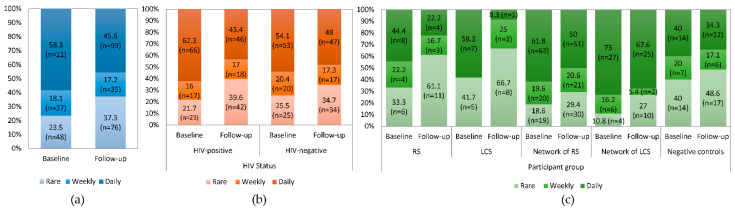
Frequencies of the responses to questionnaire Q3 (How often did you inject drugs? This includes occasions when someone else injected you) (*n* = 204), at baseline and follow-up of Transmission Reduction Intervention Project (TRIP) (**a**) all participants, (**b**) by human immunodeficiency virus (HIV) status, and (**c**) by participant group. Abbreviations: RS, recent seeds; LCS, control seeds with long-term HIV infection.

The number of different people who the participants injected drugs with did not change significantly between baseline and follow-up [median: 4 (2–7.5) at baseline and 3.5 (1–10) at follow-up (*p* = 0.896)] (Table S6). Similar results were found in the subgroup analysis by HIV status or participant group. Nonetheless, the frequency of drug injection overall decreased at follow-up compared to baseline (*p* < 0.001) as demonstrated in Figure 2a. Significantly fewer HIV-positive participants (*p* = 0.008) reported that they injected drugs daily (*n* = 46, 43.4%) during the past six months compared to baseline (*n* = 66, 62.3%) (Figure 2b). The subgroup analysis for participant groups showed safer behaviors at follow-up but the results were not significant (*p* > 0.05) (Figure 2c).

The proportion of the time the PWID shared, gave injection equipment (syringe, cooker, filter, rinse water) or backloaded (piggy-back) to inject drugs decreased at follow-up compared to the baseline of TRIP (Tables S7–S11). There was an overall significant increase of adopting safer behaviors at follow-up regarding sharing syringes (from 65.5%, *n* = 133 to 76.4%, *n* = 155; *p* = 0.007), cooker, filter or rinse water (from 38.6%, *n* = 78 to 62.4%, *n* = 126; *p* < 0.001) and backload (piggy-back) (from 74.3%, *n* = 150 to 89.1%, *n* = 180; *p* < 0.001) (Supplementary Materials, Tables S7, S9 and S11). This was evident both among the HIV-positive and negative PWID. Statistically significant increase of PWID adopting safer behaviors were also found among the RS and the network of RS, while for the latter the increase of safer behaviors was significant for all three sharing equipment practices. Furthermore, the percentage of giving someone a syringe decreased from 37.1% (*n* = 75) to 24.3% (*n* = 49) (*p* = 0.002) and from 54% (*n* = 109) to 36.6% (*n* = 74) for giving someone cookers, filters or rinse water syringes or cookers, filters or rinse water (*p* < 0.001) (Supplementary Materials, Tables S8 and S10).

Multivariable logistic regression models produced significant relationships between all drug injection-related behaviors at follow-up and baseline. Sharing or giving a syringe was related to age and backloading with gender (Table 2 and Table S12). Older people were less likely to share (aOR: 0.936, 95%CI: 0.887, 0.988) or give someone (aOR: 0.947, 95%CI: 0.898, 0.999) syringes than younger participants and females were more likely to backload to share injection drugs (aOR: 2.934, 95% CL: 1.014, 8.489) than men.

Univariable analysis did not produce any significant results for the improvement of drug injection-related behavior at follow-up (Table S13). Yet after adjusting for participant group, females were less likely to improve their behavior related to sharing cookers, filters or rinse water (aOR: 0.273, 95% CI: 0.100, 0.745) than men.

### 3.4. Sexual Behaviors

The analysis was performed only for heterosexual sex partners since the number of homosexual partners was not sufficient to carry out a statistical analysis. Medians with interquartile range and means with standard deviation of the proportions of sex partners who the participants had safe sex with are presented in Table 3. Overall, there was an increase in having safer sex, with males’ mean proportion increasing from 48 ± 46% to 59 ± 47% (*p* value = 0.023) while for women it increased from 42 ± 45% to 49 ± 45% (*p* = 0.417). The improvement among male participants was more profound among the HIV-positives (55 ± 47% versus 75 ± 43%; *p* = 0.035) and the network member of RS (36 ± 44% versus 58 ± 47%; *p* = 0.006). HIV-positives showed significant increase in having safe sex (55 ± 47 versus 75 ± 43, *p* = 0.035). This was also found for the subgroup of HIV-positives who continued drug injection at follow-up (Supplementary Materials, Table S14).

Univariable fractional regression models suggested that homeless people (β = 0.012, 95% CI: 0.001–0.023, males; β = 0.015, 95% CI: 0.009–0.021, females) and post high school educated (β = 0.018, 95% CI: 0.007–0.030) or HIV-positive (β = 0.061, 95% CI: 0.040–0.082) males were more likely to have safe sex compared to non-homeless people and up to high school educated or HIV-negative males (Table 4). These results were not supported by multivariable fractional regression models except for homeless females (β = 0.014 95% CI: 0.004, 0.024). Moreover, being a male RS, led to a significantly higher condom use (β = 0.514, 95% CI: 0.406, 0.622) when compared to being a male, member of the HIV-negative group.

## 4. Discussion

This study examined behavioral changes observed among people who inject drugs and participated in a network-based intervention in Athens, Greece. A high percentage of TRIP participants at baseline were recruited also at follow-up. Overall, the results were encouraging, yielding statistically significant changes approximately six months after enrollment in the study. Precisely, at follow-up, injecting drug use decreased and some drug injection-related behaviors such as sharing of or borrowing injecting equipment became less prevalent.

Age was a significant predictor of some injecting practices. Increasing age was independently associated with decreased odds of sharing injecting equipment and remained significant for the sharing of syringes after adjustments. Older age has previously been associated with decreased risk for sharing needles and overall healthier injecting practices [23,24]. Given that PWID are commonly introduced into injecting by others, one reason for the association between younger age and sharing may be that younger PWID still rely on others’ in helping them injecting. In this study, older age was also found to be associated with the improvement in the frequency of drug injection at follow-up versus baseline. All these stress the need to intensify prevention efforts for young PWID.

After adjusting for other predictors, employment status was associated with drug injection and frequency of injecting drugs. The multivariable regression model revealed that employed participants were about two times more likely to stop drug injection at follow-up compared to the unemployed. These results agree with previous studies highlighting that chronic drug use [25] and daily heroin injection [26] were strongly negatively associated with legal employment. Social programs to increase employment of PWID should be considered in prevention planning.

Female PWID were less likely than males to adopt some safer behaviors. These findings are in line with previous studies that suggest that females are more likely to engage in needle borrowing, ancillary equipment sharing, and being injected by someone else [27,28]. Additionally, it has been reported that females were more likely than males to report recent sexual intercourse and to have PWID sex partners [28]. Additionally, young women often have experienced a shorter duration of illicit drug use prior to initiation to injecting [29] and initiate injecting at an earlier age than their young male counterparts. It seems that young females may experience an accelerated progression of behaviors, as they compound often their risk by injecting and having sex with risk partners, and are under increased normative pressure by people in their networks [30,31]. All these hinder the education about viral transmission and decrease the likelihood of adoption and maintenance of safe behaviors [32]. Alike other settings, in Greece interventions focusing on women to reduce sexual and injection practices that put them at risk for contracting HIV are needed.

This study has some limitations. First of all, the numbers in certain participant groups (i.e., recent seeds, control seeds with Long-term HIV infection) were possibly not adequate for powerful statistical analysis. This most definitely has affected the results and could be the reason for not reaching a significant statistical level in certain instances. Secondly, the analysis included only participants who turned up and responded to questions at follow-up. Certain groups of people (like the homeless) pose a greater challenge to track down and keep in touch with [33]. Bias is likely if those that did not respond or lost to follow-up could have behaved differently than those who were followed-up. Finally, bias could also be introduced because of social desirability and the possibility of participants wanting to give the impression that their behavior improved by participating in the program.

For purposes of future research, a more advanced analytical approach, such as social network analysis, could allow researchers to depict important relations among the various participants, which might reveal unexplored ways of how behaviors can change within a network-based intervention [34]. The longitudinal structure was a big advantage of this analysis, nevertheless, since TRIP was not a targeted behavioral change intervention, modifications of the study design based on behavior theory are needed in the future.

Behavioral change, especially amongst recently HIV-infected and therefore highly infective individuals, is a key determinant of the containment of HIV. If behavioral changes are also accompanied by ART treatment, they can reduce the risk of transmission drastically [35,36]. Maintenance of behavior change for long periods of time is also very important. Interventions targeting behavior change including network-based approaches should facilitate access to HIV counseling and testing, and to the national services for drug use. Developing relationships between the different organizations that come into contact with PWID and training staff members in how to work with PWID are also important to establishing successful prevention strategies. Clinicians should also be informed of how alerting and training PWID and their network members could reduce risky behaviors and ultimately HIV transmission. Moreover, they could assist by engaging and recruiting PWID into TRIP-like interventions.

## 5. Conclusions

TRIP focused on recently HIV-infected PWID and on people of their drug injection or sexual networks. Safer drug injection-related and sexual behaviors were observed at follow-up compared to the baseline. It seems that a network-based, enhanced contact tracing approach as that of TRIP could also have a positive effect on behavior change and consequently on reducing HIV transmission. TRIP was a highly targeted program, beneficial primarily for key populations, such as PWID. Of course, it could also be of use and considered in settings with widespread transmission in the general population (generalized HIV epidemics).

## Figures and Tables

**Table 1:** Sociodemographic characteristics of Transmission Reduction Intervention Project (TRIP) participants (*n* = 292) who were followed up with six months after their recruitment.

Sociodemographic Characteristics		Participant Groups [*n* (%)]					Total	*p* Value
		RS	LCS	Network of RS	Network of LCS	Negative Controls		
Overall		22 (7.5)	17 (5.8)	136 (46.6)	47 (16.1)	70 (24.0)	292	_
Gender	Male	17 (77.3)	14 (82.4)	106 (77.9)	37 (78.7)	57 (81.4)	231 (79.1)	0.974
	Female	5 (22.7)	3 (17.6)	30 (22.1)	10 (21.3)	13 (18.6)	61 (20.9)	
Age	median (IQR)	39.5 (31–44)	36 (32–40)	35 (30–39)	34 (31–37)	36 (32–45)	35 (31–41)	0.161
Nationality	Greek	20 (90.9)	15 (88.2)	123 (90.4)	42 (89.4)	69 (98.6)	269 (92.1)	0.246
	Non-Greek	2 (9.1)	2 (11.8)	13 (9.6)	5 (10.6)	1 (1.4)	23 (7.9)	
Education	Up-to high school	19 (86.4)	15 (88.2)	115 (84.6)	39 (83.0)	62 (88.6)	250 (85.6)	0.911
	Post high School	3 (13.6)	2 (11.8)	21 (15.4)	8 (17.0)	8 (11.4)	42 (14.4)	
Employment ^1^	Employed	3 (13.6)	2 (11.8)	23 (16.9)	12 (25.5)	20 (28.6)	60 (20.5)	0.197
	Unemployed	19 (86.4)	15 (88.2)	113 (83.1)	35 (74.5)	50 (71.4)	232 (79.5)	
Homelessness ^1^	Homeless	2 (9.1)	2 (11.8)	24 (17.8)	9 (19.2)	2 (2.9)	39 (13.4)	*0.031*
	Non-homeless	20 (90.9)	15 (88.2)	111 (82.2)	38 (80.8)	68 (97.1)	252 (86.6)	
HIV status	Positive	22 (100)	17 (100)	57 (41.9)	26 (55.3)	0 (0)	122 (41.8)	*<0.001*
	Negative	0 (0)	0 (0)	79 (58.1)	21 (44.7)	70 (100)	170 (58.2)	

The Chi-squared test was used to test the difference in the frequencies and the Kruskal–Wallis equality-of-populations rank test was used to test a difference in the age distribution. *p* Values in italics stand for statistically significant differences between groups (*p* < 0.05). ^1^ Employment and homelessness status at the time of follow-up. Abbreviations: HIV, human immunodeficiency virus; IQR, interquartile range; RS, recent seeds; LCS, control seeds with long-term HIV infection.

**Table 2:** Odds ratios (OR) and 95% confidence intervals (CI) of univariable and multivariable binary logistic regression models for participants responses to questionnaire items of drug injection-related behaviors (safe versus risky) at follow up [Q4: When you injected drugs, what proportion of the time did you share a syringe that someone else had previously used to inject? (Q4a), share a cooker, filter or rinse water that someone else had previously used to inject? (Q4c)].

Factors		Drug Injection-Related Behaviors in Follow-up			
		Q4a		Q4c	
		Univariable	Multivariable	Univariable	Multivariable
Baseline response		**3.767**	**3.237**	**2.929**	**2.530**
		**(1.919–7.392)**	**(1.595–6.569)**	**(1.554–5.521)**	**(1.304–4.909)**
Gender (Females vs. males)		2.151	-	1.561	-
		(0.996–4.646)		(0.761–3.199)	
Age		**0.923**	**0.936**	**0.946**	0.961
		**(0.878–0.971)**	**(0.887–0.988)**	**(0.908–0.985)**	(0.919–1.004)
Education (Post vs. up to high School)		0.553	-	1.190	-
		(0.182–1.687)		(0.522–2.715)	
Employment (Unemployed vs. employed)		1.150	-	1.919	-
		(0.439–3.011)		(0.776–4.747)	
Homelessness ^1^ (Homeless vs. non-homeless)		1.046	-	1.048	-
		(0.439–2.490)		(0.492–2.232)	
HIV status (Positive vs. negative)		1.167	-	1.021	-
		(0.612–2.226)		(0.583–1.788)	
Participant group (Reference group: Negative controls)	RS	1.143	0.881	0.577	0.483
		(0.286–4.570)	(0.200–3.878)	(0.168–2.930)	(0.133–1.749)
	LCS	1.200	1.073	0.750	0.883
		(0.259–5.559)	(0.211–5.464)	(0.189–2.974)	(0.206–3.794)
	Network of RS	0.920	0.657	0.699	0.615
		(0.352–2.402)	(0.234–1.845)	(0.317–1.538)	(0.267–1.417)
	Network of LCS	2.333	1.648	1.853	1.334
		(0.810–6.725)	(0.532–5.101)	(0.731–4.700)	(0.499–1.417)

Odds ratios of variables whose confidence intervals do not include 1 are shown in bold. Abbreviations: HIV, human immunodeficiency virus; RS, recent seeds; LCS, control seeds with long-term HIV infection.

**Table 3:** Proportions of heterosexual partners who the participants of TRIP had safe sex with during the past six months.

SociodemographicCharacteristics		Women, Men Always Used Condom with			Men, Women Always Used Condom with		
		[Median (%); (IQR)/Mean ± SD]			[Median (%); (IQR)/Mean ± SD]]		
		Baseline	Follow-up	*p* Value	Baseline	Follow-up	*p* Value
Overall		50 (0–100)/	100 (0–100)/	*0.019*	25 (0–97)/	50 (0–100)/	0.939
		48 ± 46	59 ± 47	*0.023*	42 ± 45	49 ± 45	0.417
HIV status	Positive	73 (0–100)/	100 (46–100)/	*0.026*	95 (0–100)/	83 (0–100)/	0.462
		55 ± 47	75 ± 43	*0.035*	62 ± 46	58 ± 45	0.727
	Negative	45 (0–100)/	67 (0–100)/	0.188	0 (0–55)/	23 (0–100)/	0.472
		45 ± 46	52 ± 47	0.195	28 ± 38	41 ± 45	0.209
Participant group	RS	97 (0–100)/	100 (100–100)/	*0.048*	0 (0–0)/	25 (0–75)/	0.162
		57 ± 53	100 ± 0	0.074	0 ± 0	38 ± 48	0.215
	LCS	0 (0–100)/	0 (0–100)/	1.000	75 (50–100)/	50 (0–100)/	0.317
		40 ± 55	40 ± 55	1.000	75 ± 35	50 ± 71	0.500
	Network of RS	0 (0–100)/	88 (0–100)/	*0.007*	21 (0–96)/	33 (0–100)/	0.825
		36 ± 44	58 ± 47	*0.006*	43 ± 46	47 ± 49	0.703
	Network of LCS	100 (0–100)/	100 (0–100)/	0.774	97 (0–100)/	70 (0–98)/	0.181
		63 ± 45	65 ± 46)	0.885	63 ± 48	56 ± 45	0.694
	Negative controls	67 (0–100)/	73 (0–100)/	0.491	25 (0–60)/	50 (0–100)/	0.671
		57 ± 45	52 ± 48)	0.292	34 ± 38	50 ± 41	0.344

The Wilcoxon signed-rank test was used to test the difference of the matched pairs at baseline versus follow-up. *p* Values in italics stand for statistically significant differences between groups (*p* < 0.05). Abbreviations: HIV, human immunodeficiency virus; IQR, Interquartile range; SD, standard deviation; RS, recent seeds; LCS, control seeds with long-term HIV infection.

**Table 4:** Regression coefficients and 95% confidence intervals (CI) of univariable and multivariable fractional regression models for proportions of sex partners who heterosexual participants of TRIP had safe sex with.

Factors		Sexual Behavior			
		Women, Who Men Always Used Condom with		Men, Who Women Always Used Condom with	
		Univariable Analysis	Multivariable Analysis	Univariable Analysis	Multivariable Analysis
Baseline response		0.153	0.16	0.13	0.13
		(0.108–0.197)	(0.114–0.207)	(0.032–0.228)	(0.022–0.239)
Age		−0.088	-	−0.684	−0.402
		(−0.428–0.252)		(−1.301–−0.066)	(−1.095–0.292)
Education (Post vs. up-to high School)		0.018	0.013	−0.025	-
		(0.007–0.030	(−0.007–0.033)	(−0.094–0.044)	
Employment (Unemployed vs. employed)		0.099	-	−0.018	-
		(−0.011–0.210)		(−0.289–0.254)	
Homelessness (Homeless vs. non-homeless)		0.012	0.006	0.015	0.014
		(0.001–0.023	(−0.013–0.025)	(0.009–0.021)	(0.004–0.024)
HIV Positive (Positive vs. negative)		0.061	-	0.069	-
		(0.040–0.082)		(−0.035–0.173)	
Participant group (Reference group: Negative controls)	RS	0.472	0.514	−0.075	−0.023
		(0.330–0.614)	(0.406–0.622)	(−0.549–0.398)	(−0.479–0.432)
	LCS	−0.128	−0.056	0.05	−0.113
		(−0.582–0.326)	(−0.470–0.295)	(−0.689–0.788)	(−0.651–0.425)
	Network of RS	0.062	0.133	0.021	−0.053
		(−0.120–0.244)	(−0.030–0.295)	(−0.284–0.325)	(−0.335–0.230)
	Network of LCS	0.109	0.102	0.053	−0.161
		(−0.130–0.347)	(−0.123–0.326)	(−0.308–0.414)	(−0.548–0.227)

Fractional response regression modelling for the proportion of safe sexual partners. Data is presented as marginal increase in the overall proportion with 95% confidence intervals (CI). Abbreviations: HIV, human immunodeficiency virus; RS, recent seeds; LCS, control seeds with long-term HIV infection.

## Data Availability

The data presented in this study may be available on request from the corresponding author.

## Supplementary Materials

The following are available online at https://www.mdpi.com/1660-4601/18/5/2388/s1, Figure S1: Combination of questions for proportions of heterosexual sex partners who the participants of TRIP had safe sex with during the past six months, Table S1: Questionnaire used for the interviews in the Transmission Reduction Intervention Project, Table S2: Sociodemographic characteristics of the participants of TRIP (*n* = 357) who were followed-up and lost to follow-up six months after their recruitment, Table S3: Frequencies and percentages of the responses to questionnaire item Q1 (Have you injected drugs, or had someone other than an accredited professional inject you with any drugs?) (*n* = 292), at baseline and follow-up of TRIP, Table S4: Sociodemographic characteristics of people who injected drugs at baseline of TRIP (*n* = 270). Comparison of those who stopped injecting and continued injecting drugs at follow-up, Table S5: Odds ratios (OR) and 95% Confidence Intervals (CI) for injecting drug use at follow-up (A) (all participants) and (B) for stopping injecting drug use at follow-up (participant who injected at baseline)—TRIP, Table S6: Median values and interquartile ranges (IQR) of the number of different people who the participants injected drugs with, at baseline and follow-up of TRIP, Table S7: Frequencies and percentages (in parentheses) of the responses to questionnaire item Q4a: When you injected drugs, what proportion of the time did you share a syringe someone else had previously used to inject? (*n* = 203) at baseline and at follow-up of TRIP, Table S8: Frequencies and percentages (in parentheses) of the responses to questionnaire item Q4b: When you injected drugs, what proportion of the time did you give someone a syringe to use that you already injected with? (*n* = 202), at baseline and at follow-up of TRIP, Table S9: Frequencies and percentages (in parentheses) of the responses to questionnaire item Q4(c): When you injected drugs, what proportion of the time did you share a cooker, filter or rinse water that someone else had previously used to inject? (*n* = 202) at baseline and at follow-up of TRIP, Table S10: Frequencies and percentages (in parentheses) of the responses to questionnaire item Q4(d): When you injected drugs, what proportion of the time did you give someone a cooker, filter or rinse water that you had previously used to inject? (*n* = 202), at baseline and at follow-up of TRIP, Table S11: Frequencies and percentages (in parentheses) of the responses to questionnaire item Q4(e): When you injected drugs, what proportion of the time did you backload (piggy-back) to share injection drugs? (*n* = 202), at baseline and at follow-up of TRIP, Table S12: Odds ratios (OR) and 95% confidence intervals (CI) of univariable and multivariable logistic regression models for participants responses to questionnaire items of drug injection-related behaviors at follow-up (Q4b, Q4d, Q4e), Table S13: Odds ratios (OR) and 95% confidence intervals (CI) of univariable and multivariable logistic regression models for improvement in participants responses to questionnaire items of drug injection-related behaviors in follow-up (Q4a, Q4c, Q4e), Table S14: Proportions of women sex partners, men had safe sex with during the past six months. Data are presented as medians and means with interquartile range and standard deviation.
