# Supplementary material for: Drug Injection-Related and Sexual Behavior Changes in Drug Injecting Networks after the Transmission Reduction Intervention Project (TRIP): A Social Network-Based Study in Athens, Greece
Source: Int J Environ Res Public Health. 2021 Mar 1;18(5):2388. doi: 10.3390/ijerph18052388 (PMC7967732; doi:10.3390/ijerph18052388)
Supplement: Supplementary file 1 [file ijerph-18-02388-s001.pdf]

Supplementary material

Table S1: Questionnaire used for the interviews in the Transmission Reduction Intervention Project. All questionnaire items have reference to the period during the past six months of the interview.

**Questionnaire items (Q) and *Responses (R)***			
Drug injection status			
Q1	Have you injected drugs, or had someone other than an accredited professional inject you with any drugs?		
R1	*a) Yes*	*b) No*	
Drug injection-related behaviors			
Q2	How many different people did you inject drugs with?		
R2	*Define number: …….*		
Q3	How often did you inject drugs? This includes occasions when someone else injected you.		
R3	*a) Never*	*b) Only a few times*	*c) 1-3 times/month*
	d) About once/week	*e) 2-5 times/week*	*f) About once/day*
	g) 2 to 3 times/day, almost every day	*h) 4 to 9 times/day, almost every day*	*i) 10+ times/day, almost every day*
Q4	When you injected drugs, what proportion of the time did you:		
	a) share a syringe that someone else had previously used to inject?		
	b) give someone a syringe to use that you already injected with?		
	c) share a cooker, filter or rinse water that someone else had previously used to inject?		
	d) give someone a cooker, filter or rinse water that you had previously used to inject?		
	e) backload (piggy-back) to share injection drugs?		
R4	*a) None of the time*	*b) Very little*	*c) Less than half*
	d) About half	*e) More than half*	*f) Almost all*
	g) All	*h) Not applicable*	*i) Not asked*
	j) Does not know		
Sexual behaviors			
	For men:		
Q5	How many different women have you had vaginal or anal sex (total)?		
R5	*Define number: ……*		
Q6	How many of these women did you always use condoms with?		
R6	*Define number: ……*		
	For women:		
Q7	How many different men have you had vaginal or anal sex with?		
R7	*Define number: …….*		
Q8	How many of these men (total) did you always use condoms with?		
R8	*Define number: ……*		

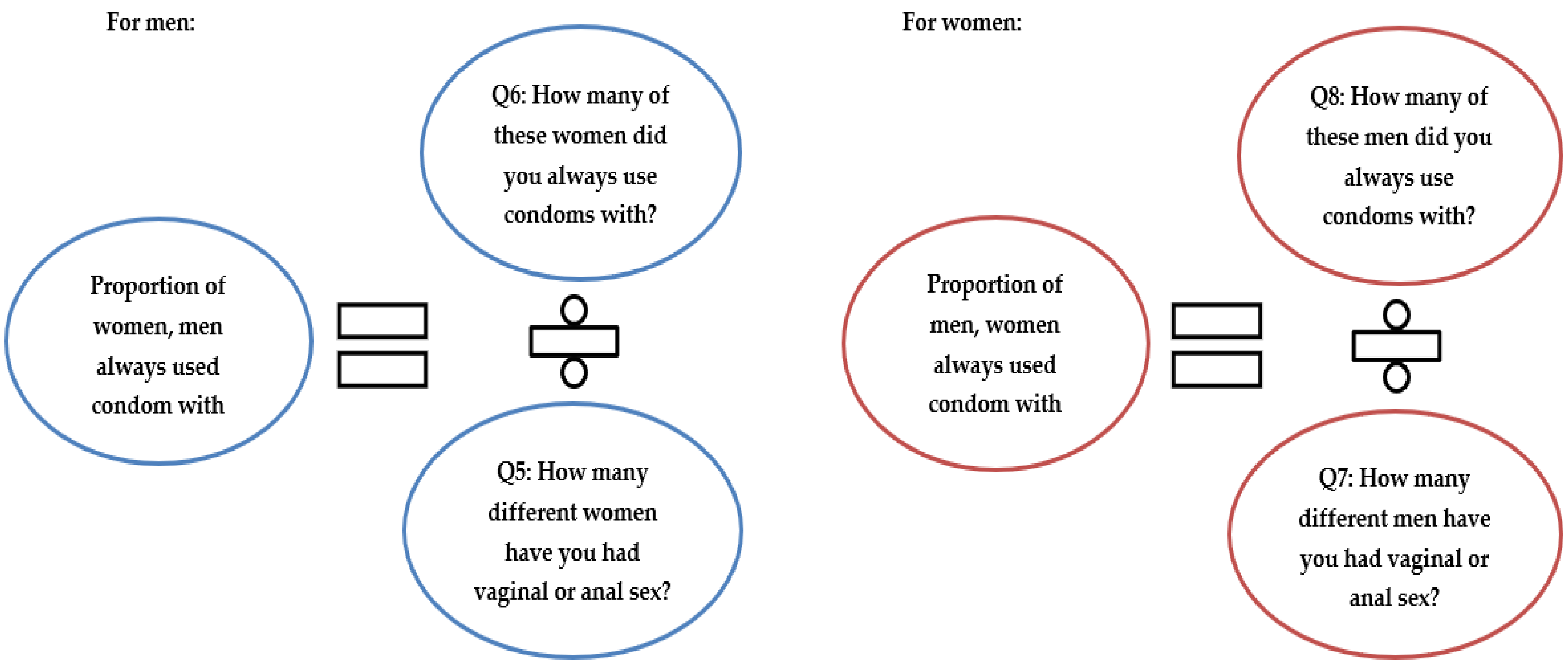

Figure S1: Combination of questions for proportions of heterosexual sex partners who the participants of TRIP (Transmission Reduction Intervention Project) had safe sex with during the past six months.

Table S2: Sociodemographic characteristics of the participants of Transmission Reduction Intervention Project (TRIP) (n=357) who were followed-up and lost to follow-up six months after their recruitment.

**Sociodemographic characteristic**		**Followed-up [n (%)]**	**Lost to follow-up [n (%)]**	**P-value**
Overall		292 (81.8)	65 (18.2)	-
Gender	Male	231 (79.1)	51 (78.5)	0.908
	Female	61 (20.9)	14 (21.5)	
Age	median (IQR)	35 (31-41)	35 (31-44)	0.751
Nationality	Greek	269 (92.1)	54 (83.1)	*0.025*
	Non-Greek	23 (7.9)	11 (16.9)	
Education	Up-to high school	249 (85.3)	60 (92.3)	0.133
	Post high School	43 (14.7)	5 (7.7)	
Employment	Employed	241 (82.5)	55 (84.6)	0.687
	Unemployed	51 (17.5)	10 (15.4)	
Homelessness	Homeless	56 (19.2)	25 (38.5)	*0.001*
	Non-homeless	235 (80.8)	40 (61.5)	
HIV status	Positive	122 (41.8)	28 (43.1)	0.848
	Negative	170 (58.2)	37 (56.9)	
Participant group	Recent Seeds (RS)	22 (7.5)	1 (1.5)	*0.030*
	Control Seeds with Long-term HIV infection (LCS)	17 (5.8)	2 (3.1)	
	Network of RS	136 (46.6)	35 (53.9)	
	Network of LCS	47 (16.1)	18 (27.7)	
	Negative controls	70 (24.0)	9 (13.9)	

The Chi-squared test was used to test the difference between frequencies and the Wilcoxon rank-sum (Mann-Whitney) test was used to test the difference in age distribution.

P-values in italics stand for statistically significant differences between groups (p<0.05).

Abbreviations: IQR, Interquartile range; SD, standard deviation; RS, recent seeds; LCS, control seeds with long-term HIV infection.

Table S3: Frequencies and percentages (in parentheses) of the responses to questionnaire item Q1 (Have you injected drugs, or had someone other than an accredited professional inject you with any drugs?) (n=292), at baseline and follow-up of Transmission Reduction Intervention Project (TRIP).

**Sociodemographic characteristics**		**Drug injection status [n (%)]**								**P-value**
		**Baseline**		**Follow up**						
		**Injecting**	**Non-injecting**	**Injecting**			**Non-injecting**			
				**Started**	**Continued**	**Total**	**Stopped**	**Continued**	**Total**	
Overall		270 (92.5)	22 (7.5)	6 (2.1)	205 (70.2)	211 (72.3)	65 (22.3)	16 (5.5)	81 (27.7)	*<0.001*
Gender	Male	217 (80.4)	14 (63.6)	6 (100)	168 (82.0)	174 (82.5)	49 (75.4)	8 (50.0)	57 (70.4)	*<0.001*
	Female	53 (19.6)	8 (36.4)	0 (0.0)	37 (18.0)	37 (17.5)	16 (24.6)	8 (50.0)	24 (29.6)	*<0.001*
Age	median (IQR)	35 (31-40)	37.5 (30-44)	38.5 (37-42)	35 (31-40)	35 (31-40)	35 (32-42)	35 (29.5-44.5)	35 (32-44)	0.279
Nationality	Greek	247 (91.5)	22 (100)	6 (100)	184 (89.8)	190 (90.1)	63 (96.9)	16 (100)	79 (97.5)	*<0.001*
	Non-Greek	23 (8.5)	0 (0.0)	0 (0.0)	21 (10.2)	9 (9.9)	2 (3.1)	0 (0.0)	2 (2.5)	*0.500*
Education	Up-to high school	234 (86.7)	16 (72.7)	6 (100)	178 (86.8)	184 (87.2)	56 (86.2)	10 (62.5)	66 (81.5)	*<0.001*
	Post high school	36 (13.3)	6 (27.3)	0 (0.0)	27 (13.2)	27 (12.8)	9 (13.8)	6 (37.5)	15 (18.5)	*0.004*
Employment[1]	Employed	47 (17.4)	13 (59.1)	1 (16.7)	28 (13.7)	29 (13.7)	19 (29.2)	12 (75.0)	31 (38.3)	*<0.001*
	Unemployed	223 (82.6)	9 (40.9)	5 (83.3)	177 (86.3)	182 (86.3)	46 (70.8)	4 (25.0)	50 (61.7)	*<0.001*
Homelessness[1]	Homeless	35 (13.0)	4 (18.2)	3 (50.0)	31 (15.2)	34 (16.2)	4 (6.1)	1 (6.3)	5 (6.2)	1.000
	Non-homeless	234 (87.0)	18 (81.8)	3 (50.0)	173 (84.8)	176 (83.8)	61 (93.9)	15 (93.8)	76 (93.8)	*<0.001*
HIV status	Positive	119 (44.1)	3 (13.6)	1 (16.7)	107 (52.2)	108 (48.8)	12 (18.5)	2 (12.5)	14 (17.3)	*0.003*
	Negative	151 (55.9)	19 (86.4)	5 (83.3)	98 (47.8)	103 (51.2)	53 (81.5)	14 (87.5)	67 (82.7)	*<0.001*
Participant group	RS	22 (8.2)	0 (0.0)	0 (0.0)	18 (8.8)	18 (8.5)	4 (6.2)	0 (0.0)	4 (4.9)	0.125
	LCS	17 (6.3)	0 (0.0)	0 (0.0)	13 (6.3)	13 (6.2)	4 (6.2)	0 (0.0)	4 (4.9)	0.125
	Network of RS	118 (43.7)	18 (81.8)	5 (83.3)	102 (49.8)	107 (50.7)	16 (24.6)	13 (81.3)	29 (35.8)	*0.027*
	Network of LCS	43 (15.9)	4 (18.2)	1 (16.7)	37 (18.0)	38 (18.0)	6 (9.2)	3 (18.7)	9 (11.1)	0.125
	Negative controls	70 (25.9)	0 (0.0)	0 (0.0)	35 (17.1)	35 (16.6)	35 (53.8)	0 (0.0)	35 (43.2)	*<0.001*

The Exact McNemar significance probability test was used to test the difference of the matched pairs in injecting *versus* non-injecting at baseline and follow-up and the Kruskal-Wallis equality-of-populations rank test was used to test a difference in the age distribution.

P-values in italics stand for statistically significant differences between groups (p<0.05).

[1] Employment and homelessness status at the time of follow-up.

Abbreviations: IQR, Interquartile range; RS, recent seeds; LCS, control seeds with long-term HIV infection.

Table S4: Sociodemographic characteristics of people who injected drug at baseline of Transmission Reduction Intervention Project (TRIP) (n=270). Comparison of those who stopped injecting and continued injecting drugs at follow-up.

**Sociodemographic characteristics**		**Drug injection status at follow-up [n (%)]**			**P-value**
		**Continued**	**Stopped**	**Total**	
Overall		205 (75.9)	65 (24.1)	270	
Gender	Male	168 (77.4)	49 (22.6)	217	0.245
	Female	37 (69.8)	16 (30.2)	53	
Age	Median (IQR)	35 (31-40)	35 (32-42)		0.056
Nationality	Greek	184 (74.5)	63 (25.5)	247	0.071
	Non-Greek	21 (91.3)	2 (8.7)	23	
Education	Up-to high school	178 (76.1)	56 (23.9)	234	0.889
	Post high School	27 (75.0)	9 (25.0)	36	
Employment	Employed	28 (59.6%)	19 (40.4%)	47	*0.004*
	Unemployed	177 (79.4)	46 (20.6)	223	
Homelessness	Homeless	31 (88.6)	4 (11.4)	35	0.059
	Non-homeless	173 (73.9)	61 (26.1)	234	
HIV status	Positive	107 (89.9)	12 (10.1)	119	*<0.001*
	Negative	98 (64.9)	53 (35.1)	151	
Participant group	RS	18 (81.8)	4 (18.2)	22	*<0.001*
	LCS	13 (76.5)	4 (23.5)	17	
	Network of RS	102 (86.4)	16 (13.6)	118	
	Network of LCS	37 (86.0)	6 (14.0)	43	
	Negative controls	35 (50.0)	35 (50.0)	70	

The Chi-squared test was used for the comparisons, except for age where a Wilcoxon rank-sum (Mann-Whitney) test was used to test the difference in age distribution.

P-values in italics stand for statistically significant differences between groups (p<0.05).

Abbreviations: IQR, Interquartile range; SD, standard deviation; RS, recent seeds; LCS, control seeds with long-term HIV infection.

Table S5: Odds ratios (OR) and 95% Confidence Intervals (CI) for injecting drug use at follow-up (A) (all participants) and (B) for stopping injecting drug use at follow-up (participant who injected at baseline) - Transmission Reduction Intervention Project (TRIP).

**Factors**		**Q1: Have you injected drugs, or had someone other than an accredited professional inject you with any drugs?**			
		**A**		**B**	
		**Univariable**	**Multivariable**	**Univariable**	**Multivariable**
Response at baseline		**8.410 (3.160-22.383)**	**10.576 (3.346-33.433)**	**-**	**-**
Gender (Females vs males)		**0.505 (0.279-0.915)**	**0.399 (0.195-0.819)**	1.482 (0.761-2.889)	**-**
Age		**0.962 (0.933-0.993)**	0.966 (0.930-1.004)	**1.043 (1.006-1.081)**	1.032 (0.991-1.074)
Education (Post vs up-to high School)		0.646 (0.324-1.289)	-	1.060 (0.470-2.386)	-
Employment (Unemployed vs employed)		**3.891 (2.146-7.056)**	**2.706 (1.332-5.495)**	**0.383 (0.197-0.746)**	**0.475 (0.228-0.988)**
Homelessness (Homeless vs non-homeless)		**2.936 (1.106-7.797)**	2.024 (0.659-6.218)	0.366 (0.124-1.079)	-
HIV status[1] (Positive vs negative)		**5.018 (2.656-9.479)**	-	**0.207 (0.105-0.411)**	-
Participant group (reference group: negative controls)	RS	4.500 (1.382-14.650)	**4.420 (1.298-15.052)**	**0.222 (0.068-0.723)**	**0.233 (0.070-0.778)**
	LCS	3.250 (0.965-10.950)	2.780 (0.791-9.773)	0.308 (0.091-1.037)	0.344 (0.100-1.175)
	Network of RS	**3.690 (1.979-6.878)**	**4.903 (2.348-10.239)**	**0.157 (0.077-0.318)**	**0.186 (0.090-0.382)**
	Network of LCS	**4.222 (1.779-10.023)**	**5.369 (1.965-14.671)**	**0.162 (0.061-0.433)**	**0.185 (0.068-0.502)**

Binary logistic regression used for the Odds Ratio (95% CI) of answering *yes* versus *no* at follow-up. Factors (except for participant group) non-significantly related to injecting drug use in univariable logistic regression analysis were excluded from multivariable analysis.

[1] HIV status was not included in the multivariable logistic regression analysis for participants who stopped injecting drug use in follow-up because of collinearity with participant group.

Odds ratios of variables whose confidence intervals do not include 1 are shown in bold.

Abbreviations: RS, recent seeds; LCS, control seeds with long-term HIV infection.

Table S6: Median values and interquartile ranges (IQR) of the number of different people who the participants injected drugs with, at baseline and follow-up of Transmission Reduction Intervention Project (TRIP).

**Q2: How many different people did you inject drugs with? (n=204)**				
		**Baseline [n (%)]**	**Follow-up [n (%)]**	**P-value**
Overall		4 (2-7.5)	3.5 (1-10)	0.896
HIV Status	Positive	4 (3-7)	4 (1-10)	0.947
	Negative	4 (2-10)	3 (1-10)	0.893
Participant group	Recent Seeds (RS)	3.5 (1-5)	4 (1-7)	0.230
	Control Seeds with Long-term HIV infection (LCS)	3 (3-7.5)	2.5 (0.5-5.5)	0.077
	Network of RS	4 (2-7)	3 (1-10)	0.611
	Network of LCS	6 (4-10)	6 (2-20)	0.826
	Negative controls	3 (1-10)	3 (1-10)	0.699

The Wilcoxon signed-rank test was used to test the difference of the matched pairs at baseline versus follow-up.
Abbreviations: RS, recent seeds; LCS, control seeds with long-term HIV infection.

Table S7: Frequencies and percentages (in parentheses) of the responses to questionnaire item Q4a: When you injected drugs, what proportion of the time did you share a syringe someone else had previously used to inject? (n=203) at baseline and at follow-up of Transmission Reduction Intervention Project (TRIP).

			Drug injection-related behavior (Q4a)		
			**Safe [n, (%)]**	**Risky [n, (%)]**	**P-value**
Overall		Baseline	133 (65.5)	70 (34.5)	*0.007*
		Follow-up	155 (76.4)	48 (23.7)	
HIV Status	Positive	Baseline	66 (62.3)	40 (37.7)	*0.029*
		Follow-up	80 (75.5)	26 (24.5)	
	Negative	Baseline	67 (69.1)	30 (30.9)	*0.169*
		Follow-up	75 (77.3)	22 (22.7)	
Participant group	Recent Seeds (RS)	Baseline	11 (61.1)	7 (38.9)	*0.250*
		Follow-up	14 (77.8)	4 (22.2)	
	Control Seeds with Long-term HIV infection (LCS)	Baseline	8 (66.7)	4 (33.3)	*1.000*
		Follow-up	9 (75.0)	3 (25.0)	
	Network of RS	Baseline	65 (64.4)	36 (35.6)	*0.007*
		Follow-up	81 (80.2)	20 (19.8)	
	Network of LCS	Baseline	21 (56.8)	16 (43.2)	*0.804*
		Follow-up	23 (62.2)	14 (37.8)	
	Negative controls	Baseline	28 (80.0)	7 (20.0)	*1.000*
		Follow-up	28 (80.0)	7 (20.0)	

The responses were collapsed into the categories, safe (none of the time) and risky (very little; less than half; about half; more than half; almost all; all) behavior. The Exact McNemar significance probability test was used to test the difference of the matched pairs in baseline versus follow-up.

P-values in italics stand for statistically significant differences between groups ($p < 0.05$).

Abbreviations: RS, recent seeds; LCS, control seeds with long-term HIV infection.

Table S8: Frequencies and percentages (in parentheses) of the responses to questionnaire item Q4b: When you injected drugs, what proportion of the time did you give someone a syringe to use that you already injected with? (n=202), at baseline and at follow-up of Transmission Reduction Intervention Project (TRIP).

			Drug injection-related behavior (Q4b)		
			**Safe [n, (%)]**	**Risky [n, (%)]**	**P-value**
Overall		Baseline	127 (62.9)	75 (37.1)	*0.002*
		Follow-up	153 (75.7)	49 (24.3)	
HIV Status	Positive	Baseline	74 (70.5)	31 (29.5)	*0.035*
		Follow-up	87 (82.9)	18 (44.4)	
	Negative	Baseline	53 (54.6)	44 (45.4)	0.035
		Follow-up	66 (68.0)	31 (32.0)	
Participant group	Recent Seeds (RS)	Baseline	12 (66.7)	6 (33.3)	0.453
		Follow-up	15 (83.3)	3 (16.7)	
	Control Seeds with Long-term HIV infection (LCS)	Baseline	9 (81.8)	2 (18.2)	1.000
		Follow-up	9 (81.8)	2 (18.2)	
	Network of RS	Baseline	59 (58.4)	42 (41.6)	*0.005*
		Follow-up	76 (75.3)	25 (24.8)	
	Network of LCS	Baseline	24 (64.9)	13 (35.1)	0.791
		Follow-up	26 (70.3)	11 (29.7)	
	Negative controls	Baseline	23 (65.7)	12 (34.3)	0.344
		Follow-up	27 (77.1)	8 (22.9)	

The responses were collapsed into the categories, safe (none of the time) and risky (very little; less than half; about half; more than half; almost all; all) behavior.

The Exact McNemar significance probability test was used to test the difference of the matched pairs in baseline versus follow-up.

P-values in italics stand for statistically significant differences between groups ($p < 0.05$).

Abbreviations: RS, recent seeds; LCS, control seeds with long-term HIV infection.

Table S9: Frequencies and percentages (in parentheses) of the responses to questionnaire item Q4(c): When you injected drugs, what proportion of the time did you share a cooker, filter or rinse water that someone else had previously used to inject? (n=202) at baseline and at follow-up of Transmission Reduction Intervention Project (TRIP).

			Drug injection-related behavior (Q4c)		
			**Safe [n, (%)]**	**Risky [n, (%)]**	**P-value**
Overall		Baseline	78 (38.6)	124 (61.4)	*<0.001*
		Follow-up	126 (62.4)	76 (37.6)	
HIV Status	Positive	Baseline	37 (35.2)	68 (64.8)	*<0.001*
		Follow-up	65 (61.9)	40 (38.1)	
	Negative	Baseline	41 (42.3)	56 (57.7)	*0.002*
		Follow-up	61 (62.9)	36 (37.1)	
Participant group	Recent seeds (RS)	Baseline	6 (33.3)	12 (66.7)	0.065
		Follow-up	13 (72.2)	5 (27.8)	
	Control Seeds with Long-term HIV infection (LCS)	Baseline	6 (54.6)	5 (45.5)	1.000
		Follow-up	7 (63.6)	4 (36.4)	
	Network of RS	Baseline	41 (40.6)	60 (59.4)	*<0.001*
		Follow-up	68 (67.3)	33 (32.7)	
	Network of LCS	Baseline	9 (24.3)	28 (75.7)	*0.039*
		Follow-up	17 (46.0)	20 (54.1)	
	Negative controls	Baseline	16 (45.7)	19 (54.3)	0.267
		Follow-up	21 (60.0)	14 (40.0)	

The responses were collapsed into the categories, safe (none of the time) and risky (very little; less than half; about half; more than half; almost all; all) behavior.

The Exact McNemar significance probability test was used to test the difference of the matched pairs in baseline versus follow-up.

P-values in italics stand for statistically significant differences between groups ($p < 0.05$).

Abbreviations: RS, recent seeds; LCS, control seeds with long-term HIV infection.

Table S10: Frequencies and percentages (in parentheses) of the responses to questionnaire item Q4(d): When you injected drugs, what proportion of the time did you give someone a cooker, filter or rinse water that you had previously used to inject? (n=202), at baseline and at follow-up of Transmission Reduction Intervention Project (TRIP).

			**Drug injection-related behavior (Q4d)**		
			**Safe [n, (%)]**	**Risky [n, (%)]**	**P-value**
Overall		Baseline	93 (46.0)	109 (54.0)	*<0.001*
		Follow-up	128 (63.4)	74 (36.6)	
HIV Status	Positive	Baseline	53 (50.5)	52 (49.5)	*0.002*
		Follow-up	74 (70.5)	31 (29.5)	
	Negative	Baseline	40 (41.2)	57 (58.8)	*0.020*
		Follow-up	54 (55.7)	43 (44.3)	
Participant group	Recent seeds (RS)	Baseline	8 (44.4)	10 (55.6)	0.070
		Follow-up	14 (77.8)	4 (22.2)	
	Control Seeds with Long-term HIV infection (LCS)	Baseline	8 (72.7)	3 (27.3)	1.000
		Follow-up	9 (81.8)	2 (18.2)	
	Network of RS	Baseline	48 (47.5)	53 (52.5)	*0.004*
		Follow-up	67 (66.3)	34 (33.7)	
	Network of LCS	Baseline	12 (32.4)	25 (67.6)	0.267
		Follow-up	17 (45.9)	20 (54.1)	
	Negative controls	Baseline	17 (48.6)	18 (51.4)	0.344
		Follow-up	21 (60.0)	14 (40.0)	

The responses were collapsed into the categories, safe (none of the time) and risky (very little; less than half; about half; more than half; almost all; all) behavior.

The Exact McNemar significance probability test was used to test the difference of the matched pairs in baseline versus follow-up.

P-values in italics stand for statistically significant differences between groups ($p < 0.05$).

Abbreviations: RS, recent seeds; LCS, control seeds with long-term HIV infection.

Table S11: Frequencies and percentages (in parentheses) of the responses to questionnaire item Q4(e): When you injected drugs, what proportion of the time did you backload (piggy-back) to share injection drugs? (n=202), at baseline and at follow-up of Transmission Reduction Intervention Project (TRIP).

			Drug injection-related behavior (Q4e)		
			**Safe [n, (%)]**	**Risky [n, (%)]**	**P-value**
Overall		Baseline	150 (74.3)	52 (25.7)	*<0.001*
		Follow-up	180 (89.1)	22 (10.9)	
HIV Status	Positive	Baseline	76 (72.4)	29 (27.6)	*0.004*
		Follow-up	92 (87.6)	13 (12.4)	
	Negative	Baseline	74 (76.3)	23 (23.7)	*0.003*
		Follow-up	88 (90.7)	9 (9.3)	
Participant group	Recent seeds (RS)	Baseline	11 (61.1)	7 (38.9)	*0.031*
		Follow-up	17 (94.4)	1 (5.6)	
	Control Seeds with Long-term HIV infection (LCS)	Baseline	8 (72.7)	3 (27.3)	0.250
		Follow-up	11 (100)	0 (0.0)	
	Network of RS	Baseline	78 (77.2)	23 (22.8)	*0.007*
		Follow-up	92 (91.1)	9 (8.9)	
	Network of LCS	Baseline	25 (67.6)	12 (32.4)	0.344
		Follow-up	29 (78.4)	8 (21.6)	
	Negative controls	Baseline	28 (80.0)	7 (20.0)	0.375
		Follow-up	31 (88.6)	4 (11.4)	

The responses were collapsed into the categories, safe (none of the time) and risky (very little; less than half; about half; more than half; almost all; all) behavior. The Exact McNemar significance probability test was used to test the difference of the matched pairs in baseline versus follow-up.

P-values in italics stand for statistically significant differences between groups ($p < 0.05$).

Abbreviations: RS, recent seeds; LCS, control seeds with long-term HIV infection.

Table S12: Odds ratios (OR) and 95% confidence intervals (CI) of univariable and multivariable logistic regression models for participants responses to questionnaire items of drug injection-related behaviors (safe *versus* risky) at follow-up (Q4b, Q4d, Q4e).

		**Drug injecting related behaviors in follow-up**					
		**Q4b**		**Q4d**		**Q4e**	
**Factors**		**Univariable**	**Multivariable**	**Univariable**	**Multivariable**	**Univariable**	**Multivariable**
Baseline response		**3.373**	**2.814**	**3.583**	**2.649**	**5.222**	**4.686**
		**(1.732-6.567)**	**(1.403-5.647)**	**(1.926-6.669)**	**(1.358-5.168)**	**(2.079-13.116)**	**(1.723-12.743)**
Gender		1.705	-	1.435	-	**3.133**	**2.934**
(Females vs males)		(0.784-3.708)		(0.697-2.953)		**(1.206-8.138)**	**(1.014-8.489)**
Age		**0.924**	**0.947**	**0.927**	**0.933**	**0.921**	0.956
		**(0.879-0.971)**	**(0.898-0.999)**	**(0.888-0.968)**	**(0.888-0.980)**	**(0.859-0.988)**	(0.884-1.033)
Education		0.903	-	1.043	-	0.297	-
(Post vs up-to high School)		(0.343-2.378)		(0.451-2.409)		(0.038-2.301)	
Employment		2.029	-	**2.942**	**4.247**	1.605	-
(Unemployed vs employed)		(0.669-6.158)		**(1.070-8.094)**	**(1.391-12.972)**	(0.354-7.275)	
Homelessness		1.177	-	0.946	-	1.602	-
(Homeless vs non-homeless)		(0.509-2.721)		(0.439-2.038)		(0.548-4.685)	
HIV status		**0.449**	-	0.526	-	1.444	-
(Positive vs negative)		**(0.233-0.865)**		(0.297-0.931)		(0.589-3.541)	
Participant group (Negative controls as reference group)	RS	0.675	0.590	0.429	0.256	0.456	0.291
		(0.155-2.934)	(0.124-2.816)	(0.117-1.574)	(0.060-1.085)	(0.047-4.412)	(0.027-3.144)

LCS	0.675	0.830	0.300	0.283	-	-
	(0.122-3.736)	(0.138-5.000)	(0.057-1.581)	(0.048-1.682)		
Network of RS	1.083	0.906	0.729	0.472	0.712	0.656
	(0.439-2.676)	(0.347-2.362)	(0.332-1.603)	(0.191-1.170)	(0.205-2.472)	(0.172-2.494)
Network of LCS	1.375	1.265	1.853	1.193	2.067	1.629
	(0.479-3.951)	(0.415-3.857)	(0.731-4.700)	(0.429-3.317)	(0.563-7.591)	(0.392-6.778)

Q4: When you injected drugs, what proportion of the time did you (Q4b) give someone a syringe to use that you already injected with? (Q4d), give someone a cooker, filter or rinse water that you had previously used to inject? (Q4e), backload (piggy-back) to share injection drugs?

Factors (except for participant group) non-significantly related to injecting drug use in univariable logistic regression analysis were excluded from multivariable analysis.

Odds ratios of variables whose confidence intervals do not include 1 are shown in bold.

Abbreviations: RS, recent seeds; LCS, control seeds with long-term HIV infection.

Table S13: Odds ratios (OR) and 95% confidence intervals (CI) of univariable and multivariable logistic regression models for improvement in participants responses to questionnaire items of drug injection-related behaviors (safe *versus* risky) in follow-up (Q4a, Q4c, Q4e).

**Factors**		**Improvement of drug injection-related behaviors at follow-up**					
		**Q4a**		**Q4c**		**Q4e**	
		**Univariable**	**Multivariable**	**Univariable**	**Multivariable**	**Univariable**	**Multivariable**
Gender		0.728	-	**0.278**	**0.273**	0.626	**-**
(Females vs males)		(0.281-1.883)		**(0.103-0.752)**	**(0.100-0.745)**	(0.226-1.731)	
Age		0.965	**-**	0.968	**-**	**0.925**	**0.923**
		(0.919-1.014)		(0.928-1.009)		**(0.875-0.977)**	**(0.871-0.976)**
Education		1.175	-	1.105	-	1.658	-
(Post vs up-to high School)		(0.440-3.140)		(0.464-2.631)		(0.643-4.276)	
Employment		2.390	-	0.468	-	1.061	-
(Unemployed vs employed)		(0.685-8.336)		(0.206-1.063)		(0.375-3.004)	
Homelessness		0.721	-	0.969	-	1.560	-
(Homeless vs non-homeless)		(0.259-2.010)		(0.428-2.197)		(0.638-3.812)	
HIV status		1.452	-	1.165	-	1.247	-
(Positive vs negative)		(0.729-2.894)		(0.646-2.101)		(0.617-2.521)	
Participant group	RS	2.133	2.133	2.889	2.997	3.875	4.013
(Reference group: Negative controls)		(0.384-11.840)	(0.384-11.840)	(0.874-9.544)	(0.884-10.164)	(0.927-16.191)	(0.912-17.659)
	LCS	3.556	3.556	1.083	1.098	2.906	2.776
		(0.610-20.727)	(0.610-20.727)	(0.235-4.994)	(0.233-5.176)	(0.538-15.698)	(0.499-15.436)

Network of RS	3.325 (0.935-11.827)	3.325 (0.935-11.827)	1.532 (0.647-3.627)	1.565 (0.653-3.753)	1.796 (0.566-5.698)	1.509 (0.464-4.912)
Network of LCS	3.429 (0.844-13.926)	3.429 (0.844-13.926)	1.070 (0.375-3.055)	1.092 (0.377-3.167)	1.808 (0.480-6.817)	1.483 (0.382-5.751)

Q4: When you injected drugs, what proportion of the time did you share a syringe that someone else had previously used to inject? (Q4a), share a cooker, filter or rinse water that someone else had previously used to inject? (Q4c), backload (piggy-back) to share injection drugs? (Q4e).

Factors (except for participant group) non-significantly related to injecting drug use in univariable analysis were excluded from multivariable analysis.

Odds ratios of variables whose confidence intervals do not include 1 are shown in bold.

Abbreviations: RS, recent seeds; LCS, control seeds with long-term HIV infection.

Table S14: Proportions of women sex partners, men had *safe* sex with during the past six months.

Sociodemographic characteristics		Women, men always used condom with [median (%); (IQR) / mean ± SD]								
		**Continued injecting in follow-up (n=86)**			**Stopped injecting in follow-up (n=30)**			**Not injecting at baseline (n=10)**		
		**Baseline**	**Follow-up**	**P-value**	**Baseline**	**Follow-up**	**P-value**	**Baseline**	**Follow-up**	**P-value**
Overall		50 (0-100) /	100 (0-100) /	0.053	67 (0-100) /	95 (0-100) /	0.837	0 (0-0) /	58 (0-100) /	0.094
		49 ± 47	59 ± 47	0.054	60 ± 43	61 ± 46	0.933	10 ± 32	49 ± 45	0.086
HIV Status	Positive	58 (0-100) /	100 (46-100) /	*0.025*	100 (100-100) /	100 (100-100) /	-	0 (0-0) /	0 (0-0) /	-
		53 ± 47	75 ± 43	*0.035*	100 ± 0	100 ± 0	-	0 ±.	0 ±.	-
	Negative	42 (0-100) /	50 (0-100) /	0.607	67 (0-100) /	80 (0-100) /	0.845	0 (0-0) /	67 (0-100) /	0.104
		46 ± 47	50 ± 48	0.543	55 ± 43	56 ± 47	0.934	11 ± 33	55 ± 44	0.086
Participant group	Recent seeds (RS)	48 (0-100) /	100 (100-100) /	*0.050*	100 (100-100) /	100 (100-100) /	-	-	-	-
		49 ± 54	100 ± 0	0.071	100 ±.	100 ±.	-	-	-	-
	Control seeds with long-term	0 (0-50) /	0 (0-50) /	1.000	100 (100-100) /	100 (100-100) /	-	-	-	-
	HIV infection (LCS)	25 ± 50	25 ± 50	1.000	100 ±.	100 ±.	-	-	-	-
	Network of RS	50 (0-100) /	100 (0-100) /	0.078	0 (0-100) /	100 (0-100) /	0.085	0 (0-0) /	50 (0-75) /	0.161
		44 ± 44	60 ± 47	0.066	29 ± 49	62 ± 49	0.111	11 ± 33	44 ± 44	0.169
	Network of LCS	100 (0-100) /	100 (0-100) /	0.883	75 (25-100) /	75 (25-100) /	1.000	0 (0-0) /	100 (100-100) /	0.317
		68 ± 45	63 ± 48	0.649	63 ± 48	63 ± 48	1.000	0 ±.	100 ±.	-
	Negative controls	33 (0-100) /	33 (0-100) /	1.000	67 (67-100) /	80 (0-100) /	0.326	-	-	-
		49 ± 50	49 ± 48	0.981	68 ± 37	55 ± 48	0.168			

The Wilcoxon signed-rank test was used to test the difference of the matched pairs at baseline versus follow-up.

Abbreviations: IQR, Interquartile range; SD, standard deviation; RS, recent seeds; LCS, control seeds with long-term HIV infection.
